# JIA-like in a boy with Ataxia-telangiectasia

**DOI:** 10.1186/1546-0096-12-S1-P212

**Published:** 2014-09-17

**Authors:** Filipa Furtado, Ana Isabel Cordeiro, João Neves, Marta Conde

**Affiliations:** 1 Pediatric Rheumatology Unit, Hospital Dona Estefânia - CHLC, Lisbon, Portugal; 2 Primary Immunodeficiencies Unit, Hospital Dona Estefânia - CHLC, Lisbon, Portugal

## Introduction

Mechanisms of autoimmunity in primary immunodeficiencies (PID) are, in many situations, unclear. Despite being linked with many PIDs (namely humoral), juvenile idiopathic arthritis (JIA) has rarely been associated with DNA-repair disorders, such as Ataxia-telangiectasia (A-T).

## Objectives

We report a case of JIA-like in a male patient with A-T.

## Methods

A 3 year-old boy with A-T presented with pain and swelling of fourth metatarsophalangeal (MTP) joint of left foot. Six months later, this symptom persists and arthritis of the left knee was noted, so he was referred to rheumatology consult.

## Results

At first evaluation he had arthritis of both knees, left ankle and 4th MTF of the left foot and tenosynovitis of second finger of the right hand. His immune evaluation revealed an important CD4 naif lymphopenia, abnormal proliferative responses to mitogens, skewed Vbeta repertoire and expanded gammadelta T cells, as well as conserved humoral response. Laboratory investigations showed erythrocyte sedimentation rate 25 mm/hr, C- reactive protein 14.5 mg/l, antinuclear antibodies positive (1/80), anti-cyclic citrullinated protein antibodies and rheumatoid factor were negative. A JIA-like disease was assumed, he started ibuprofen (30 mg/kg/day), and a chemical synovectomy with triamcinolone hexacetonide (TH) was performed (knees and ankle).

Ten months after diagnosis, knees and left ankle remain without active synovitis, but active 4th MTP of left foot and tenosynovitis of 2nd finger of right hand persists despite moderate improvement. Taking in account the underlying immune defect methotrexate was not an option and hydroxichloroquine (5 mg/kg/day) was started.

## Conclusion

This case seems to be the first known pediatric patient with A-T who developed chronic, JIA-like disease. The management of these patients is particularly difficult because they are extremely susceptible to DNA damage and show an unusual susceptibility to viral infections (namely herpetic). These features are very important when considering the best therapeutic options for JIA-like disease.

## Disclosure of interest

None declared.

